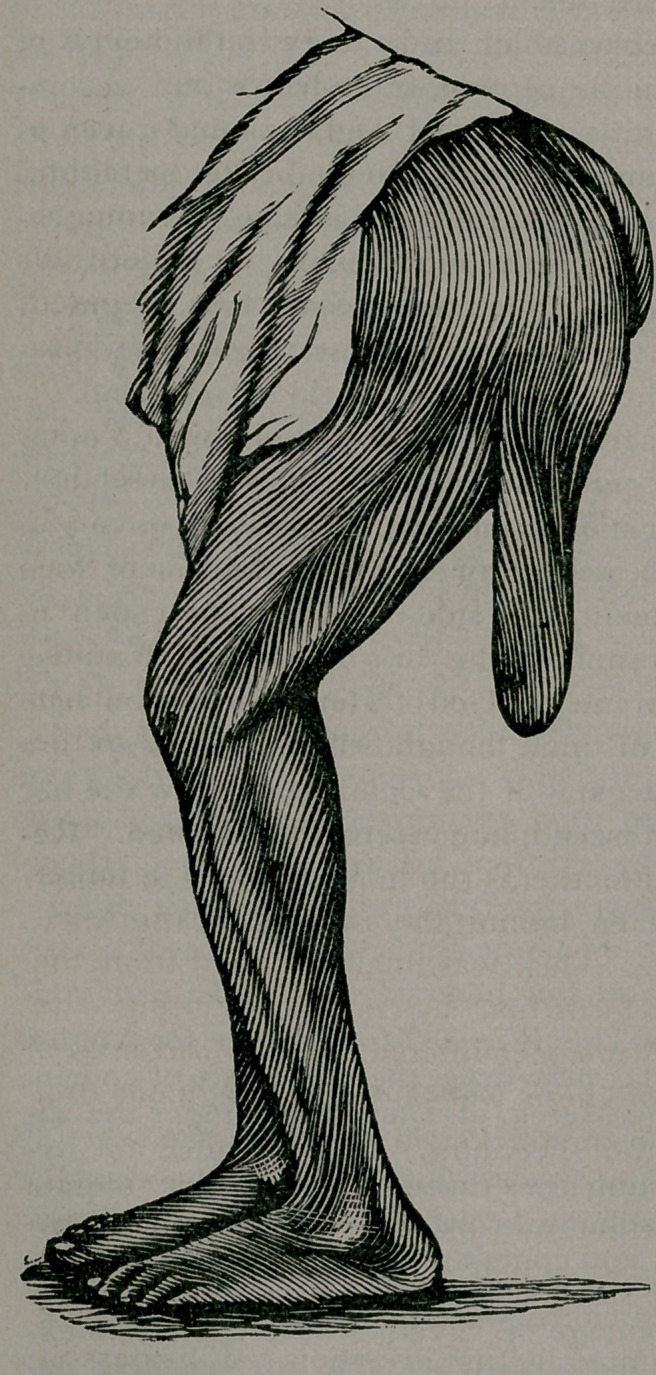# A Remarkable Case of Fibro-Cellular and Cartilaginous Tumor

**Published:** 1883-12-20

**Authors:** Jno. Thad. Johnson

**Affiliations:** Professor of Principles and Practice of Surgery in the Southern Medical College, Atlanta, Georgia


### A REMARKABLE CASE OF FIBRO-CELLULAR AND CARTILAGINOUS TUMOR.

By Jno. Thad. Johnson, M D.,
**Professor of Principles and Practice of Surgery in the Southern Medical College, Atlanta, Georgia.**

The case of which a cut is here presented, is probably of sufficient interest to justify me in placing it before the profession.

The tumor was removed October 17th, 1883, at my surgical clinic at the Southern Medical College, the patient having been admitted to the Infirmary connected with the college.

The patient is a negro man twenty-one years of age. The tumor, then very small, was first discovered when he was ten years of age. Its growth, after that time, was slow but steady. It was never attended by pain,further than that of an occasional superficial inflammation, set up by irritation of clothing, exercise, or sitting.

The tumor hung well down toward the knee; and furnishes a very good imitation of a tail, as if to give body and strength to the imagination of the Darwinites. It was flattened out very similar in shape to the tail of a beaver. The transverse diameter varied from 5 to 7 inches. Its thickness (antero-posterior) was about, or nearly, three inches in the upper portion, one and a half in the central, and somewhat thicker as we approached the extremity. Measuring around the tumor in its longest (vertical) diameter, gave us thirty-three inches. The weight of the portion removed lacked a fraction of four pounds.

The growth was cartilaginous and fibro-cellular. Occupying the most of the upper third or half of the sac (or bag), was an aggregation of irregular cartilaginous nodules. To external manipulation these well-similated a fœtus of about four months ; and the similarity to the touch at once suggested itself to all who examined it. More than one prominent physician (for the patient had "run the rounds") suspected that there might be therein the product of an imperfect twin conception, or monstrosity.

Extending upward from this nodular cartilagenous mass, was a cylindrical process of the same structure, which, in the operation, was finally traced in its attachment to the sacro-ischiatic ligaments. Before incising, it seemed to be firmly adherent to the tuberosity of the ischium itself. It also gave me some reason to be prepared for an extension between and beyond the sciatic ligaments into the bony pelvis.

The tegumentary part of the tumor partook somewhat, in its physical characteristics, of elephantiasis. A closer dissection showed it be fibro-cellular. The connective tissue was much thickened, with the fibrous elements largely increased. There was but little adipose development, though the lower part of the growth suggested, under manipulation, such a character. A number of fibrous growths were interspersed throughout the cellular hypertrophy. These were distinct, though firmly attached to the integument and cellular tissue.

In the removal, an incision was made encircling the mass three inches below the gluteal surface. Turning the flaps up, a rather tedious effort was made toward tracing the cartilaginous rope already spoken of, to its origin. This was finally severed from the margin of the sacro-ischiatic formation by combined cutting and tearing. No large vessels were encountered, though several required the ligature. One of these was in the cartilaginous pro. longation ; the others were in the thickened cellular tissue.

After ample time for arresting the oozing of the smaller vessels, the flaps were brought together by silk and wire, supported by plaster. The line of union was, when on the stretch, nearly 14 inches in length. Carbolized cloths were placed over all. About one-half of the cut surface united by first intention. Considerable suppuration occurred in the inner half of the flap. This portion of the skin extended toward and was continuous with the scrotum, and was very much hypertrophied. Free drainage was provided for. The patient progressed to a cure without serious symptoms.

We may remark on the unusual *length* of the tumor. There was no weight at the extremity to drag it to so remarkable an extent. The heavy part of the growth, indeed, was above. This cellular extension seemed to grow downward, of its own accord, as naturally as a horse's tail would grow. There was no constricted pedicle at the base.

I must ask here to acknowledge the very efficient assistance, in the operation, of Professors Roy and Nicolson and Dr. Divine, and Mr. Butler, with possibly other students of the class.

December 8th. I am now able to report that the wound healed kindly. Before union was completed, however, the integument adjacent to the line of incision began to thicken, or take on hypertrophy, very rapidly. Indeed, this growth, while not subjected to the microscope, seemed to be a true elephantiasis ; more marked, even, was the resemblance than it was before the operation, as above described. This growth soon attained the size of two small fists. On the date just given I have cut away the redundant integument as nearly as possible ; but I very much fear even this removal will not put a stop to the abnormal growth of skin.

In this second operation I could not find any recurrence whatever of the cartilaginous structure ; nor was there any renewal of a distinct fibroid mass either in the skin or cellular tissue ; but, instead of the normal integument, an hypertrophied mass of nearly two inches thickness.

*Atlanta, Ga., November, 1883.*

## Figures and Tables

**Figure f1:**